# Plasma Membrane Ca^2+^ ATPase Activity Enables Sustained Store-operated Ca^2+^ Entry in the Absence of a Bulk Cytosolic Ca^2+^ Rise

**DOI:** 10.1093/function/zqac040

**Published:** 2022-08-24

**Authors:** Pradeep Barak, Suneet Kaur, Erica Scappini, Charles J Tucker, Anant B Parekh

**Affiliations:** Department of Physiology, Anatomy and Genetics, Oxford University, Oxford OX1 3PT, UK; Oxford Nanoimaging, Linacre House, Jordan Hill Business Park Banbury Road, Oxford OX2 8TA, UK; Laboratory of Signal Transduction, National Institute of Environmental Health Sciences, NIH, Research Triangle Park NC 27709, USA; Laboratory of Signal Transduction, National Institute of Environmental Health Sciences, NIH, Research Triangle Park NC 27709, USA; Laboratory of Signal Transduction, National Institute of Environmental Health Sciences, NIH, Research Triangle Park NC 27709, USA; Department of Physiology, Anatomy and Genetics, Oxford University, Oxford OX1 3PT, UK; Laboratory of Signal Transduction, National Institute of Environmental Health Sciences, NIH, Research Triangle Park NC 27709, USA

**Keywords:** Ca^2+^, plasma membrane ATPase, calcium channel, Transcription factor

## Abstract

In many cell types, the rise in cytosolic Ca^2+^ due to opening of Ca^2+^ release-activated Ca^2+^ (CRAC) channels drives a plethora of responses, including secretion, motility, energy production, and gene expression. The amplitude and time course of the cytosolic Ca^2+^ rise is shaped by the rates of Ca^2+^ entry into and removal from the cytosol. However, an extended bulk Ca^2+^ rise is toxic to cells. Here, we show that the plasma membrane Ca^2+^ ATPase (PMCA) pump plays a major role in preventing a prolonged cytosolic Ca^2+^ signal following CRAC channel activation. Ca^2+^ entry through CRAC channels leads to a sustained sub-plasmalemmal Ca^2+^ rise but bulk Ca^2+^ is kept low by the activity of PMCA4b. Despite the low cytosolic Ca^2+^, membrane permeability to Ca^2+^ is still elevated and Ca^2+^ continues to enter through CRAC channels. Ca^2+^-dependent NFAT activation, driven by Ca^2+^ nanodomains near the open channels, is maintained despite the return of bulk Ca^2+^ to near pre-stimulation levels. Our data reveal a central role for PMCA4b in determining the pattern of a functional Ca^2+^ signal and in sharpening local Ca^2+^ gradients near CRAC channels, whilst protecting cells from a toxic Ca^2+^ overload.

## Graphical Abstract

**Graphical Abstract:**
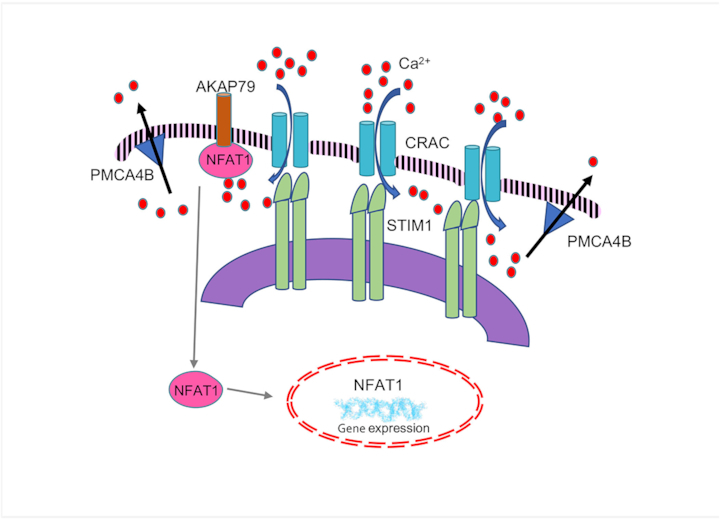

## Introduction

A rise in cytosolic Ca^2+^ is a universal trigger for activation of a remarkable breadth of cellular responses, including neurotransmitter release, contraction, energy production, gene transcription, and cell death.^1^ The dynamics of a cytosolic Ca^2+^ signal is determined by the balance between Ca^2+^ entry into the cytosol, typically accomplished through opening Ca^2+^-permeable ion channels that populate organelles and the plasma membrane, mobile and immobile Ca^2+^ buffers, and Ca^2+^ removal mechanisms that sequestrate Ca^2+^ into internal Ca^2+^ stores or eject the ion from the cell.^2^

In many cell types, particularly non-excitable cells, Ca^2+^ release-activated Ca^2+^ (CRAC) channels play a central and often indispensable role in providing the Ca^2+^ rise that drives important biological outputs.^3,4^ Stimulation of cell-surface receptors that couple either to Gq-type heterotrimeric G proteins or tyrosine kinases activate isoforms of phospholipase C to produce the second messenger inositol-1, 4, 5-trisphosphate (InsP_3_).^5^ InsP_3_ binds to and opens InsP_3_-gated Ca^2+^ channels in the endoplasmic reticulum (ER).^6^ Ca^2+^ depletion from the ER activates two proteins in the ER membrane, stromal interaction molecule 1 (STIM1) and STIM2.^7,8^ Upon Ca^2+^ store emptying, the proteins form oligomers and migrate towards the plasma membrane, where they bind to, cluster and gate open plasmalemmal Orai proteins, the pore-forming subunits of CRAC channels.^9–11^ Ca^2+^ entry through the channels drives a plethora of spatially and temporally distinct responses, including secretion, ER store refilling, and recruitment of Ca^2+^-regulated transcription factors such as c-fos, the nuclear factor of activated T cells (NFAT), nuclear factor kappa-light-chain enhancer of activated B cells (NF-κB) and cAMP response element-binding protein (CREB).^12,13^

Ca^2+^ entry through CRAC channels is buffered by mitochondria, taken into the ER by SERCA pumps or ejected from the cell by plasma membrane Ca^2+^ ATPases (PMCA) and, to a lesser extent in non-excitable cells, by the electrogenic Na^+^–Ca^2+^ exchanger. Both SERCA pumps and mitochondrial Ca^2+^ buffering have important and well-characterized consequences on the duration of CRAC channel activity. By refilling stores, SERCA pumps deactivate CRAC channels and thereby terminate Ca^2+^ entry.^14–16^ Mitochondria prolong CRAC channel activity because Ca^2+^ uptake by the mitochondrial Ca^2+^ uniporter prevents bulk cytosolic Ca^2+^ from increasing sufficiently or long enough to evoke Ca^2+^-dependent slow inactivation of the channels.^17–21^ By contrast, the role of PMCA in shaping Ca^2+^ signals generated by CRAC channels is less well understood. Four genes encode mammalian PMCAs (PMCA1–4) and alternative splicing generates more than twenty variants.^22^ The pumps are all activated by Ca^2+^-calmodulin but differ in their rates of activation and in their affinities for calmodulin.^23^

Pászty and colleagues have examined the impact of overexpression of various PMCAs on the time course of the cytosolic Ca^2+^ signal following CRAC channel activation in HeLa cells.^24^ They demonstrated that different PMCAs dramatically altered the pattern and time course of the Ca^2+^ signal in a manner dependent on how quickly the PMCA was activated by Ca^2+^-calmodulin. In the presence of the fast PMCa2b isoform, cytosolic Ca^2+^ rapidly returned to resting levels whereas slow, oscillatory signals were seen when the slower PMCA4b protein was present instead. These results clearly establish the impact of PMCAs on Ca^2+^ signals evoked by store-operated Ca^2+^ entry.

In Jurkat T lymphocytes, local Ca^2+^ entry through CRAC channels stimulates PMCA activity in two distinct phases: an initial rapid increase is followed by a form of modulation that develops over tens of seconds and enhances pump activity ∼4-fold.^25^ This slow increase in PMCA activity after store depletion gradually reduces cytosolic Ca^2+^ and ensures a stable cytosolic Ca^2+^ elevation is reached. Findings that are contradictory to this have been recently reported, also extracted from Jurkat T cells.^26^ In this latter study, STIM1 was found to inhibit PMCA, substantially delaying recovery of cytosolic Ca^2+^ by several tens of seconds following Ca^2+^ entry through CRAC channels. By prolonging the cytosolic Ca^2+^ rise, inhibition of Ca^2+^ clearance was proposed to be essential for Ca^2+^-dependent NFAT activation.^26^ A further twist has recently been provided by the same authors who found that STIM1 inhibition was prevented by the protein partner of STIM1 (POST).^27^ In the presence of overexpressed POST, PMCA activity was not blocked by STIM1 in Jurkat T cells. Cytosolic Ca^2+^ now rapidly recovered and it was suggested that the rapid decline of cytosolic Ca^2+^ was essential for NFAT activity, ostensibly by preventing Ca^2+^-dependent inactivation of CRAC channels.^27^

In this study, we have investigated the role of PMCA in shaping cytosolic Ca^2+^ signals generated by CRAC channels. Extending the findings of Bautista and Lewis,^28^ we find that modulation of the PMCA4b isoform leads to a remarkable increase in PMCA activity such that the pump more than matches Ca^2+^ entry through CRAC channels, returning cytosolic Ca^2+^ to pre-stimulation levels despite maintained channel activity. This increase in PMCA activity prevents toxic consequences of a sustained a larger cytosolic Ca^2+^ rise. By using a Ca^2+^ sensor tagged directly to Orai1, we reveal that local Ca^2+^ near the channels remains high despite bulk cytosolic Ca^2+^ declining rapidly. Finally, we demonstrate that gene expression continues unabated despite the loss of the bulk Ca^2+^ rise. Our results show that enhanced PMCA activity sharpens spatial Ca^2+^ gradients emanating from CRAC channels, increasing the signalling power of local Ca^2+^ signals. Our data reinforce the importance of Ca^2+^ nanodomains near CRAC and not a bulk Ca^2+^ rise in driving excitation–transcription coupling.

## Materials and Methods

### Cell Culture

HEK293 were purchased from ATCC (via the United Kingdom supplier LGC) and were cultured in Dulbecco's modified Eagle's medium (DMEM) (Thermo Scientific). Media were supplemented with 10% fetal bovine serum and 1% penicillin-streptomycin.

### Plasmid Constructs and Transfection

Plasmids: PMCA4b-cherry was a kind gift from Dr Agnes Enyedi. Orai1-GECO and cytosolic GECO were purchased from Addgene. NFAT1-cherry was provided by Dr Irene Frischauf (Linz University, Austria). STIM1-YFP, Orai1-GFP, and Orai1-CFP were provided by Dr James Putney (NIEHS, USA). HEK293 cells were transfected with Lipofectamine 2000 (Invitrogen) using 1 μg plasmid, and then incubated in media without penicillin-streptomycin. In the GECO experiments, only 0.6 μg plasmid was used to reduce the levels of overexpression. Experiments were then carried out 24 to 48 h after transfection.

### Cytosolic Ca^2+^ Measurements

**Figure 1.**
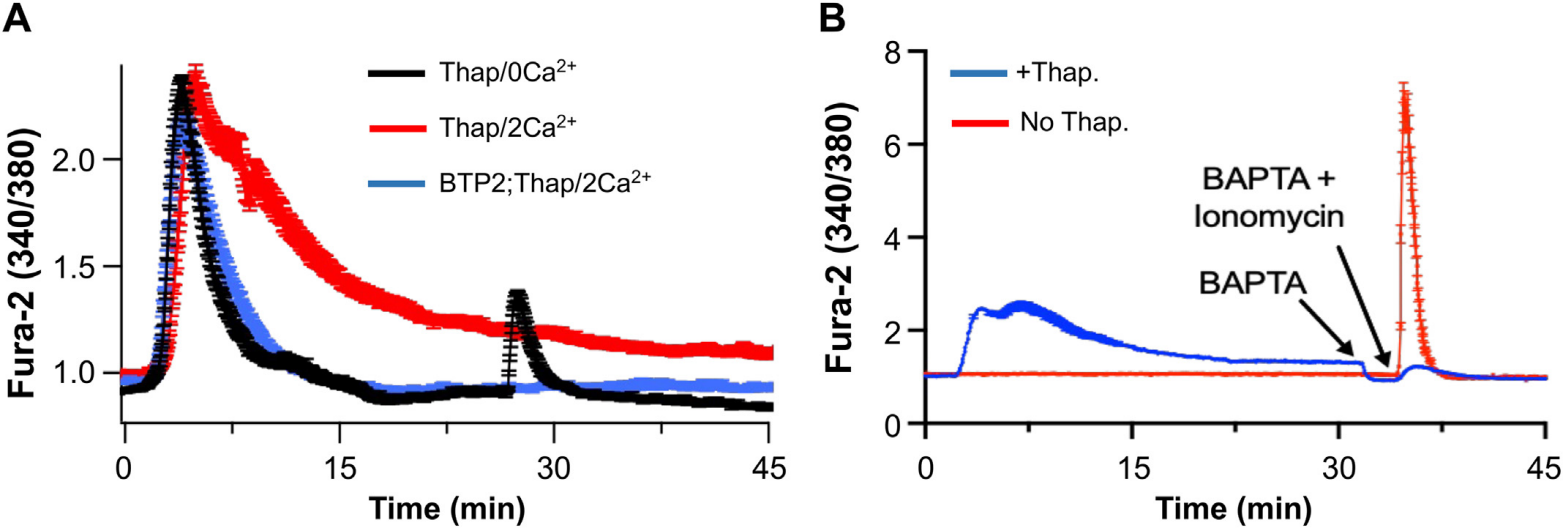
Bulk cytosolic Ca^2+^ recovers despite prolonged exposure to store depletion. (A) Time courses of cytosolic Ca^2+^ are compared for cells challenged with thapsigarin in the absence of external Ca^2+^, presence of external Ca^2+^, and following pretreatment with the channel blocker BTP2 in the presence of external Ca^2+^. Each trace is the mean ± SEM of between 29 and 30 cells. In the thapsigargin/0Ca^2+^ experiment, 1 μm ionomycin was applied at 26 min. (B) Store-operated Ca^2+^ entry evoked by thapsigargin does not refill the store, as gauged by the loss of the ionomycin response in Ca^2+^-free solution. Data are mean ± SEM of between 26 and 28 cells.

**Figure 2.**
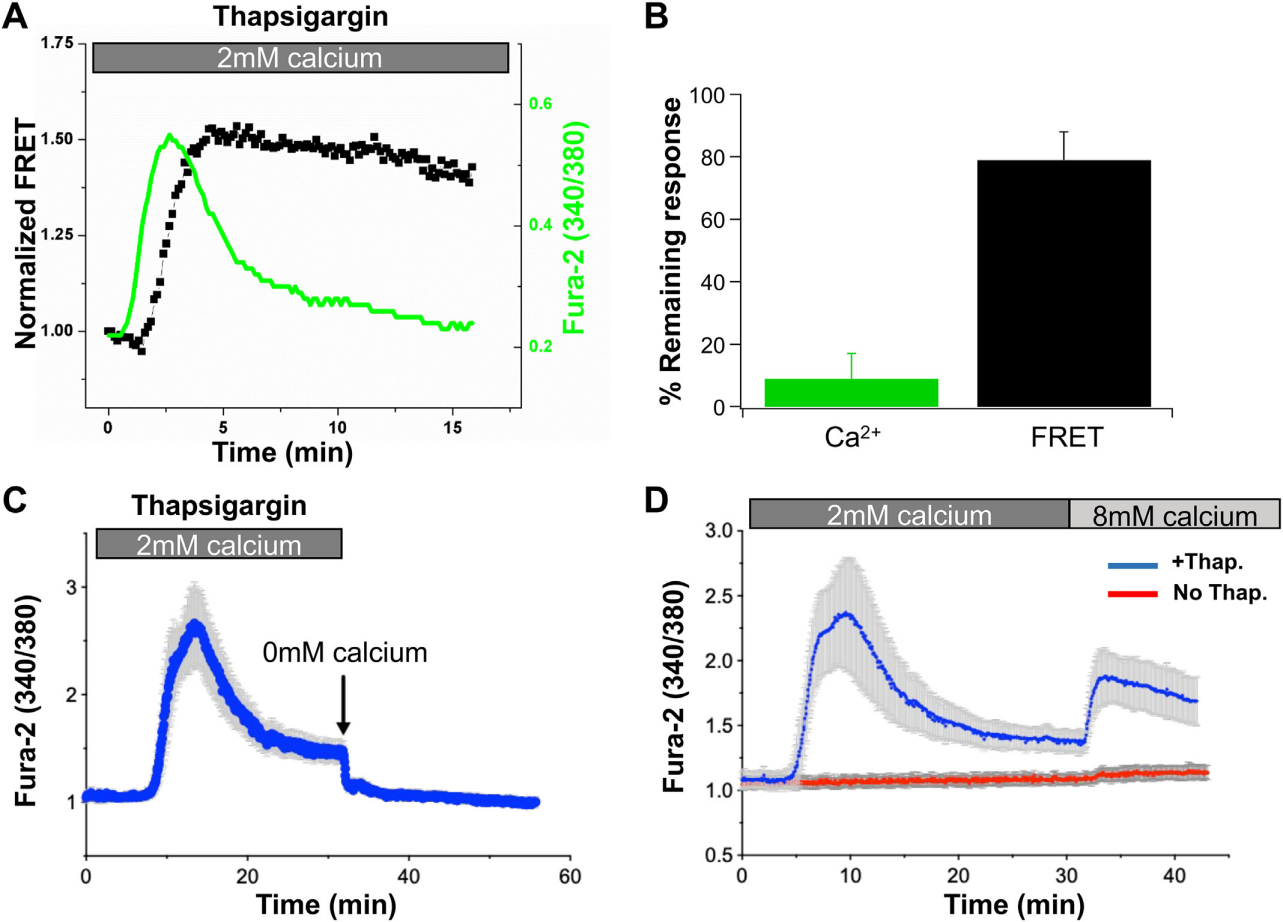
CRAC channels remain active during sustained stimulation with thapsigargin. (A) FRET signal between STIM1-YFP and Orai1-CFP was sustained (black trace) despite cytosolic Ca^2+^ recovering to resting levels (green trace). (B) Aggregate data from 4 independent experiments as in panel A are compared. *P* < 0.01 between the two groups. (C) Cytosolic Ca^2+^ falls rapidly when cells are perfused in Ca^2+^-free solution as shown. Data are mean ± SEM of 29 cells. (D) Raising external Ca^2+^ to 10 m m increases cytosolic Ca^2+^ in cells exposed to thapsigargin despite cytosolic Ca^2+^ having returned close to resting levels (blue trace). Elevating external Ca^2+^ in cells with replete stores did not increase cytosolic Ca^2+^ (red trace). Data are mean ± SEM of 27 cells (blue trace) and 27 cells (red trace).

Cytosolic Ca^2+^ was measured as previously described.^29^ Cells were loaded with 1 μm Fura 2-am for 40 min in the dark at room temperature, washed three times and then kept in the dark for a further 15 min to allow for dye de-esterification prior to recording. Fura-2 fluorescence was measured by alternately exciting the dye at 340 and 380 nm, and emission was collected at 510 nm. Changes in Ca^2+^ concentration are presented as the ratio =  *F*_340_/*F*_380_. Some experiments were carried in Oxford using an Olympus BX51 × 1 upright microscope with the commercial xcellence rt software. Data shown for Figures 1, 2C and D, 5, and 6A were obtained in the NIEHS using a Nikon TS-100 inverted microscope equipped with a 20 × fluor objective (0.75 NA). Fluorescence images of the cells were recorded and analyzed with a digital fluorescence imaging system (InCyt Im2, Intracellular Imaging Inc., Cincinnati, OH, USA) equipped with a light-sensitive CCD camera (Cooke PixelFly, ASI, Eugene, OR, USA).

### TIRF Measurements

Fluorescence images were captured on an Andor Dragonfly 505 multi modal confocal system (Oxford Instruments, Abingdon, UK) in Total Internal Refection Fluorescence (TIRF) mode using a Nikon 60X Apo TIRF 60X/1.49 objective lens. The 488 nm laser line was set to 5%, TIRF mode to penetration with a depth of 100 nm while fluorescence emission was collected with a 100 ms exposure of an Andor Zyla camera. A total of 200 time series images were acquired every 10 s during each experiment. The time series images were analyzed in FIJI (ImageJ 1.53q) where individual cells were cropped and then mean fluorescence intensity was measured above a threshold of 200 for all images.

### Airyscan Confocal Microscopy

Airyscan confocal images were taken on a Zeiss LSM880 with Airyscan (Carl Zeiss Inc, Oberkochen, Germany) using a C-Apochromat 40X/1.2 Water DIC objective. For the red channel, a 561 nm DPSS laser at 2.0% power was used for excitation of mCherry while a long pass 570 nm filter collected the emission. For the green channel, a 488 nm ArKr laser line at 5.0% power was used for excitation of GFP while a bandpass 495–550 filter was used for collection of the emission signal. The master gain setting of the airyscan detector was held constant for all images of both channels with a setting of 800. Furthermore, all images were taken with a zoom of 3, a 1.47μs pixel dwell time, a 0.05 μm pixel size, and a physical pinhole setting of 124 μm. The imaging experiment was performed as a time series of multiple positions where a total 15 images were acquired every 90 s at each position.

Individual cells were analyzed across the time series from each position. Time lapse images of each cell were imported into Imaris 9.9 (Oxford Instruments plc, Abington, UK) licensed with the Coloc feature. Co-localization thresholds for each channel were manually set and held constant across the entire time series. The number of co-localized voxels for each time point were then exported and subsequently normalized to time 0 where a mean and SE were calculated across all replicates.

### FRET Measurements

FRET measurements between STIM1-YFP and Orai1-CFP were performed on an Olympus BX51 × 1 upright microscope equipped with 40× water immersion objective and OBS megaview CCD camera. DV2 beam splitter was used to split donor emission (CFP-480/30) and acceptor emission (YFP-535/40) channels using dichroic mirror at 505 wavelength. The donor and acceptor channels were projected on separate halves of the camera and two halves were aligned using pixel by pixel alignment using the patterned slide provided by Olympus and alignment was also reconfirmed using ImageJ. Analysis of ratiometric FRET (FRET = intensity in YFP channel/intensity in CFP channel) and FRET kinetics was calculated after subtracting background intensity using the FRET module provided with the commercial xcellence rt software by Olympus. Images were recorded with 100 ms of exposure using a cell R excitation system fitted with CFP excitation filter at 0.1 Hertz frequency with no binning.

### NFAT Translocation and Cytosolic Ca^2+^ Measurements

Cytosolic Ca^2+^ and NFAT translocation were measured at the same time by loading cells expressing NFAT1-cherry with Fluo 4-am (1 μm for 30 min). Images were taken with a Zeiss LSM880 (Carl Zeiss Inc, Oberkochen, Germany) using the 488 and 594 nm laser lines for excitation paired with emission filter settings of 499–588 nm, and 590–735 nm, respectively. A C-Apochromat 40x/1.2 water objective was used for image collection.

### Statistical Analysis

All results were expressed as means ± SD unless indicated. Two-tailed Student's *t-*test was used to compare differences between two groups in all the experiments, using GraphPad Prism and statistical significance was set at a *P*-value of < 0.05.

## Results

### Sustained Activation of CRAC Channels Leads to a Transient Rise in Cytosolic Ca^2+^

Ca^2+^ store depletion following exposure to the SERCA pump blocker thapsigargin (2 μm) in the absence of external Ca^2+^ evoked a transient rise in cytosolic Ca^2+^, which then decayed monotonically to pre-stimulation levels after ∼8 min (Figure 1A, black trace). Because store depletion leads to opening of CRAC channels, we expected a prolonged increase in cytosolic Ca^2+^ following stimulation with thapsigargin in the presence of external Ca^2+^. However, this was not the case; the Ca^2+^ signal was not sustained despite continuous exposure of thapsigargin (Figure 1A, red trace). In ∼70% of cells, cytosolic Ca^2+^ decayed gradually but continuously and returned to pre-stimulation levels only ∼2.5-fold more slowly than was the case in the absence of external Ca^2+^ [(Figure 1A), see also Figures 2A, 6B, and 7E]. In the remaining cells, cytosolic Ca^2+^ fell by ∼75% from the peak value within 10–15 min. Cells from a given preparation tended to fit into one or other of these profiles. CRAC channels were functional in the presence of external Ca^2+^ because stimulation with thapsigargin in the presence of the channel blocker BTP2 resulted in a Ca^2+^ signal that was indistinguishable from that evoked by thapsigargin in Ca^2+^-free solution (Figure 1A, blue trace).

We considered various explanations for the complete return of cytosolic Ca^2+^ to resting levels despite sustained activation of CRAC channels. It is possible that stores refilled, leading to deactivation of the channels. However, this is unlikely for three reasons. First, we used a supra-maximal concentration of thapsigargin, which irreversibly inhibits SERCA pumps over the time course of our experiments. Second, we measured store Ca^2+^ content following application of the Ca^2+^ ionopore ionomycin in Ca^2+^-free solution. Control cells not exposed to thapsigargin showed a robust rise in cytosolic Ca^2+^ following challenge with ionomycin, demonstrating stores were replete with Ca^2+^ under resting conditions (red trace in Figure 1B). Exposure to thapsigargin in Ca^2+^-free solution released Ca^2+^ from stores and subsequent stimulation with ionomycin evoked a much reduced response [Figure 1A, black trace; ionomycin was applied at 1800 s; compare response here with the ionomycin response (red trace) in Figure 1B]. Hence both thapsigargin and ionomycin target the same Ca^2+^ store. Importantly, the ionomycin response was almost abolished in cells pretreated with thapsigargin in the presence of external Ca^2+^, revealing little if any store refilling had taken place despite prolonged CRAC channel activity (Figure 1B, blue trace). Third, interaction between STIM1 and Orai1 was sustained in thapsigargin-stimulated cells in the presence of 2 m m external Ca^2+^ for up to 20 min, a time when cytosolic Ca^2+^ had returned to, or close to, pre-stimulation levels (see below).

### CRAC Channels Remain Active Despite the Recovery of Cytosolic Ca^2+^

Another explanation for why the Ca^2+^ signal is transient despite sustained store depletion is that cytosolic Ca^2+^ inactivates the channels, uncoupling STIM1 from Orai1. CRAC channels exhibit both fast and slow Ca^2+^-dependent inactivation.^4,30,31^ Fast inactivation develops over milliseconds, is controlled by Ca^2+^ binding to sites located within 3 and 4 nm of the pore and, over the physiological range of voltages encountered by a cell, accounts for up to ∼40% inhibition of CRAC channels.^32–34^ Slow inactivation develops over tens of seconds and requires a rise in bulk cytosolic Ca^2+^ to the low microMolar range.^14,35,36^ Ca^2+^-dependent inactivation of CRAC channels results in dissociation of STIM1 away from the plasma membrane, so the protein is no longer able to bind to and gate Orai1 channels.^37^ Several lines of evidence suggest Ca^2+^-dependent inactivation of CRAC channels does not account for the decline in the cytosolic Ca^2+^ signal despite sustained store depletion (Figure 1A). First, recoveries from fast and slow inactivation are complete within ∼500 ms and ∼100 s, respectively.^14,33,34^ Therefore, as cytosolic Ca^2+^ falls over tens of seconds, recovery from inactivation should occur and lead to slow oscillatory Ca^2+^ signals.^38^ This was not observed; cytosolic Ca^2+^ fell continuously with time and did not oscillate (Figure 1A). Second, we measured FRET between STIM1-YFP and Orai1-CFP following stimulation with thapsigargin in the presence of external Ca^2+^ over several minutes. The FRET signal peaked after ∼4 min and remained strong despite cytosolic Ca^2+^ signal returning to pre-stimulation levels (Figure 2A). The fractional decrease in the FRET signal at 15 min was 19± 4% even though cytosolic Ca^2+^ had declined close to basal levels (Figure 2B). The sustained FRET signal in Figure 2A and B provide additional support for the absence of store refilling under these conditions; store refilling would lead to disassociation of STIM1 and Orai1 with a subsequent loss of FRET between the two proteins. A third argument against Ca^2+^-dependent inactivation is shown in Figure 2C and D. If the decline in cytosolic Ca^2+^ in Figure 1A is due to loss of CRAC channels as a consequence of strong Ca^2+^-dependent inactivation, then membrane permeability to Ca^2+^ should be low as cytosolic Ca^2+^ declines in the continuous presence of thapsigargin. To test this, we carried out two different experiments. First, removed external Ca^2+^ once cytosolic Ca^2+^ had decayed close to pre-stimulation levels. If CRAC channels had inactivated, then cytosolic Ca^2+^ should be unaltered. However, cytosolic Ca^2+^ fell quickly, consistent with channel activity (Figure 2C). Second, we raised external Ca^2+^ to 10 m m, once cytosolic Ca^2+^ was close to resting levels. A clear increase in cytosolic Ca^2+^ occurred (Figure 2D). Raising external Ca^2+^ to 10 m m in cells not exposed to thapsigargin failed to increase cytosolic Ca^2+^ (Figure 2D, red trace). Therefore, CRAC channels remain open in the continuous presence of thapsigargin, despite bulk cytosolic Ca^2+^ returning close to pre-stimulation levels.

### Measurements of Local Ca^2+^ Near Orai1 Channels

**Figure 3.**
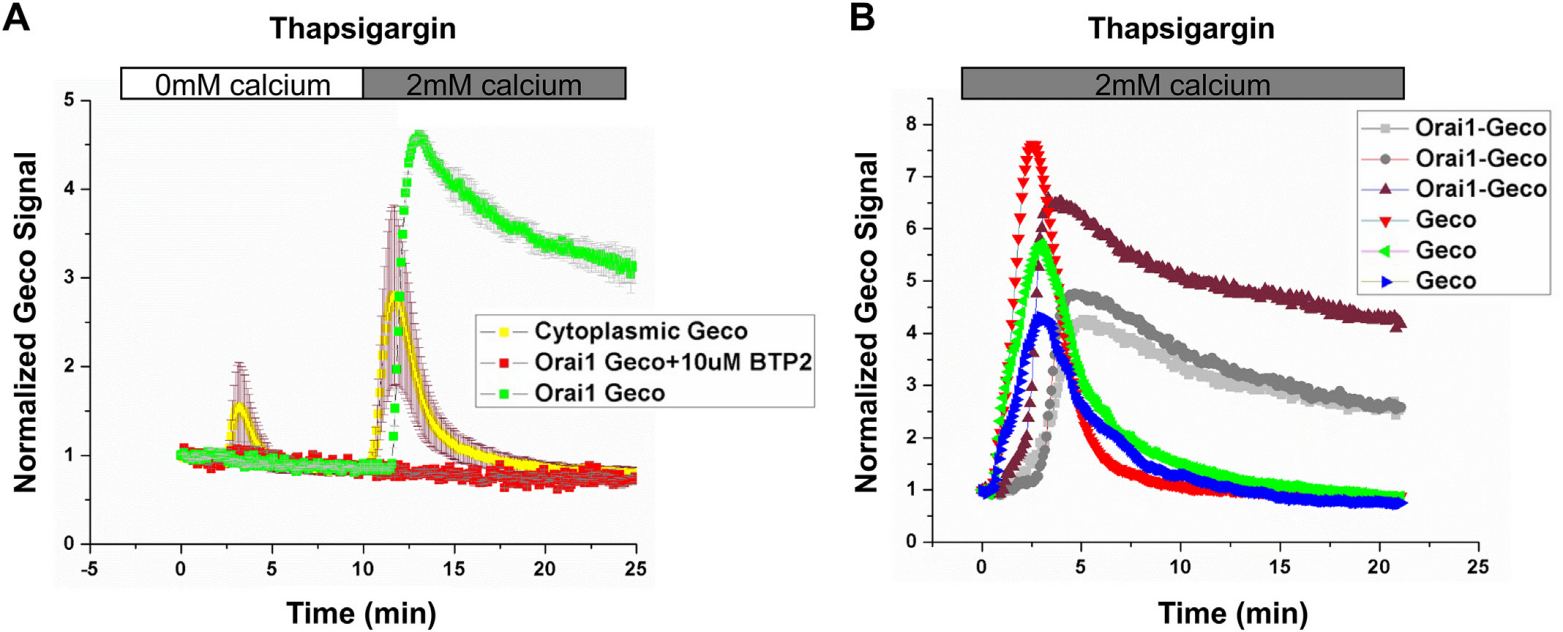
Local Ca^2+^ near CRAC channels are sustained whereas cytosolic Ca^2+^ falls quickly. (A) Traces compare Ca^2+^ signals measured using cytosolic GECO or Orai1-GECO. Cells were stimulated with thapsigargin in Ca^2+^-free solution and external Ca^2+^ was readmitted as indicated. Each trace shows the means and SEM of between 12 and 17 cells. (B) As in panel A but cells were challenged with thapsigargin in the continuous presence of external Ca^2+^. 3 cells from 3 independent experiments are shown, to demonstrate the mild variability in responses, particularly with respect to peak amplitude. Similar data were seen in a further 3 experiments.

If CRAC channels are indeed open despite bulk cytosolic Ca^2+^ being low, then local Ca^2+^ near CRAC channels should remain elevated even when the bulk cytosolic Ca^2+^ has fallen substantially. To address this directly, we expressed an Orai1 construct in which the Ca^2+^-sensitive fluorescent protein GECO had been tagged to the channel N-terminus.^39^ The GECO moiety faithfully reports Ca^2+^ close to CRAC channels and has been used to measure single Orai1 channel activity.^39^ Stimulation with thapsigargin in Ca^2+^-free solution failed to evoke a detectable Ca^2+^ rise near Orai1 channels (Figure 3A), reflecting at least in part the relatively low affinity of GECO for Ca^2+^ compared with fura 2 (∼600  versus 200 n m, respectively) and the low levels of released Ca^2+^ that reach the plasma membrane. Readmission of external Ca^2+^ resulted in a rapid rise in the Orai1-GECO signal and this decayed slowly over several minutes, falling by only ∼25% after 15 min of Ca^2+^ entry (Figure 3A, green trace). We also measured bulk Ca^2+^ by expressing GECO targeted to the cytosol. Unlike the case with Orai1-GECO, cytosolic GECO did respond to Ca^2+^ release from the stores (Figure 3A, yellow trace). Readmission of external Ca^2+^ resulted in a rise in the cytosolic GECO signal but the response was transient, fully recovering to pre-stimulation levels within 15 min (Figure 3A). No such Orai1-GECO signal was seen when BTP2 was present (Figure 3A, red trace). We repeated these experiments but stimulated cells with thapsigargin in the continuous presence of external Ca^2+^. Cytosolic GECO reported a rapid rise in cytosolic Ca^2+^, which then decayed back to pre-stimulation levels within 10–15 min (Figure 3B, labeled GECO; 3 cells from 3 independent experiments are presented to show the modest variability between experiments). Orai1-GECO responded after a delay of ∼1 min and the fluorescent signal then increased. The slower kinetics of the Orai1-GECO signal closely reflect the time taken for CRAC channels to activate following passive store depletion; CRAC current develops over several tens of seconds when store are depleted using thapsigargin. The Orai1-GECO signal remained elevated, declining by only 24± 5% after 20 min (Figure 3B). Collectively, these results confirm that CRAC channels remain open despite bulk cytosolic Ca^2+^ returning to pre-stimulation levels.

### TIRF Microscopy Measurements of Local Ca^2+^ Near CRAC Channels

**Figure 4.**
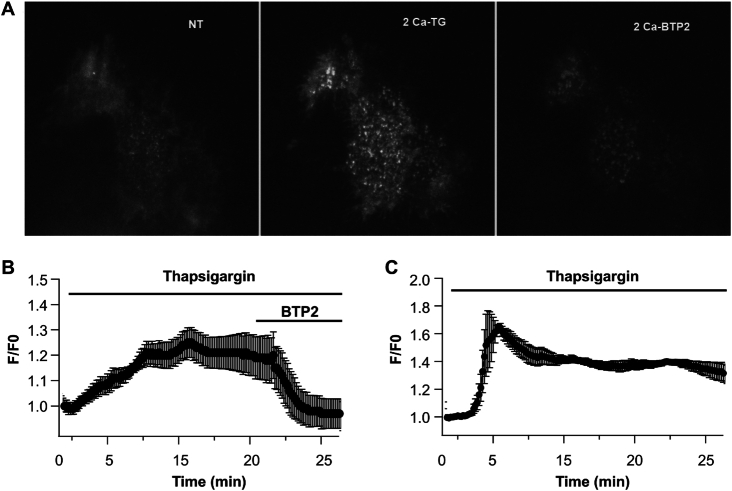
TIRF measurements of subplasmalemmal Ca^2+^ using Orai1-GECO. (A) Images compare Orai1-GECO Ca^2+^ signals under resting conditions (labeled NT for non-treated), after 20 min stimulation with thapsigargin in external Ca^2+^ (2Ca–TG) and then after the addition of 10 μm BTP2. (B) Graph depicts the time course of the Orai1-GECO signal for the treatments shown. Data are the average of 9 cells. (C) Graph shows the averaged time course of Orai1-GECO signal in EGTA-loaded cells.

We used TIRF microcopy to measure Ca^2+^ near Orai1-GECO channels, an approach that provides an opportunity to image cytosolic Ca^2+^ signals within ∼100 nm of the plasma membrane. Stimulation with thapsigargin led to a sustained rise in sub-plasmalemmal Ca^2+^ that was maintained for 20 min (Figure 4A and B). This sustained Ca^2+^ signal required Ca^2+^ influx through CRAC channels because it was abolished rapidly by the CRAC channel blocker BTP2, added after ∼20 min stimulation (Figure 4A and B). To ensure we were recording Ca^2+^ signals close to Orai1-GECO, we loaded cells with the slow chelator EGTA to restrict Ca^2+^ entry through CRAC channels to within ∼100 nm of the plasma membrane.^40,41^ In the presence of cytosolic EGTA, the local Ca^2+^ signal remained relatively sustained and was similar to that seen in non-EGTA-loaded cells (Figure 4C).

### CRAC Channels Are Functional in the Absence of a Bulk Cytosolic Ca^2+^ Rise

**Figure 5.**
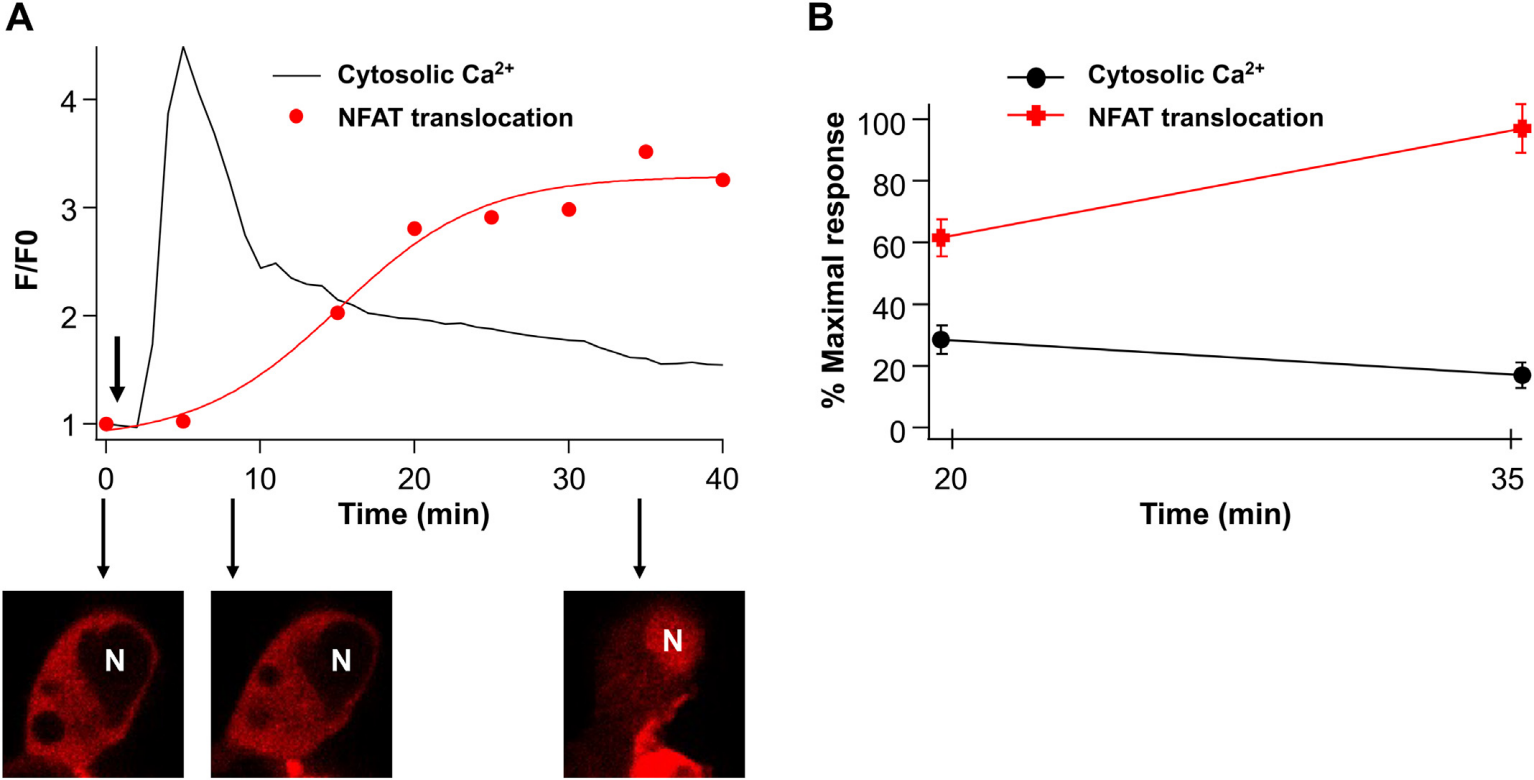
NFAT migration continues despite cytosolic Ca^2+^ being low. (A) Cytosolic Ca^2+^ and NFAT1-cherry dynamics were measured in the same cell. Images below the graph show NFAT distribution at the times indicated (*t* = 0, 6, and 35 min). Thapsigargin was applied at the arrow. N denotes nucleus. (B) Graph compares cytosolic Ca^2+^ and nuclear/cytosolic ratio of NFAT at 20 and 35 min. Both parameters have been normalized to their respective maximal responses. *P* < 0.05 between points for each condition.

We asked whether the open CRAC channels retained a functional ability to activate gene expression, despite bulk cytosolic Ca^2+^ being low. Ca^2+^ nanodomains near open CRAC channels increase expression of the immediate early gene c-fos,^42,43^ an integral component of the AP-1 transcription factor complex, and the Ca^2+^-activated transcription factor NFAT. NFAT1–4 comprise a family of Ca^2+^-dependent transcription factors, which are stimulated by the Ca^2+^-activated protein phosphatase calcineurin, the target for immunosuppressants.^44^ Local Ca^2+^ entry through CRAC channels stimulate calcineurin.^29,45^ Active calcineurin then dephosphorylates NFAT, leading to the exposure of a nuclear localization sequence, which enables NFAT to migrate into the nucleus. We measured cytosolic Ca^2+^ and NFAT1-mCherry dynamics in the same cells at the same time. Cytosolic Ca^2+^ rose to peak ∼4 min after stimulation and then declined steadily. By contrast, NFAT translocated into the nucleus considerably more slowly (Figure 5A, red trace) and continued to accumulate despite cytosolic Ca^2+^ falling substantially. After 20 min of stimulation, cytosolic Ca^2+^ had fallen by 73% (relative to the peak) and by 35 min, it had fallen by 84%. Nevertheless, between 20 and 35 min, NFAT1 translocation into the nucleus increased by 27% (Figure 5A). Aggregate data comparing cytosolic Ca^2+^ and NFAT nuclear translocation in the same cells are shown in Figure 5B. Despite cytosolic Ca^2+^ being at a relatively low level and continuing to fall, NFAT activation continued. These results are consistent with earlier work that showed loading the cytosol with EGTA to prevent a bulk cytosolic Ca^2+^ rise failed to affect the rate or extent of nuclear translocation of NFAT.^46^

### The Plasma Membrane Ca^2+^ ATPase Pump Prevents CRAC Channels From Raising Bulk Ca^2+^

**Figure 6.**
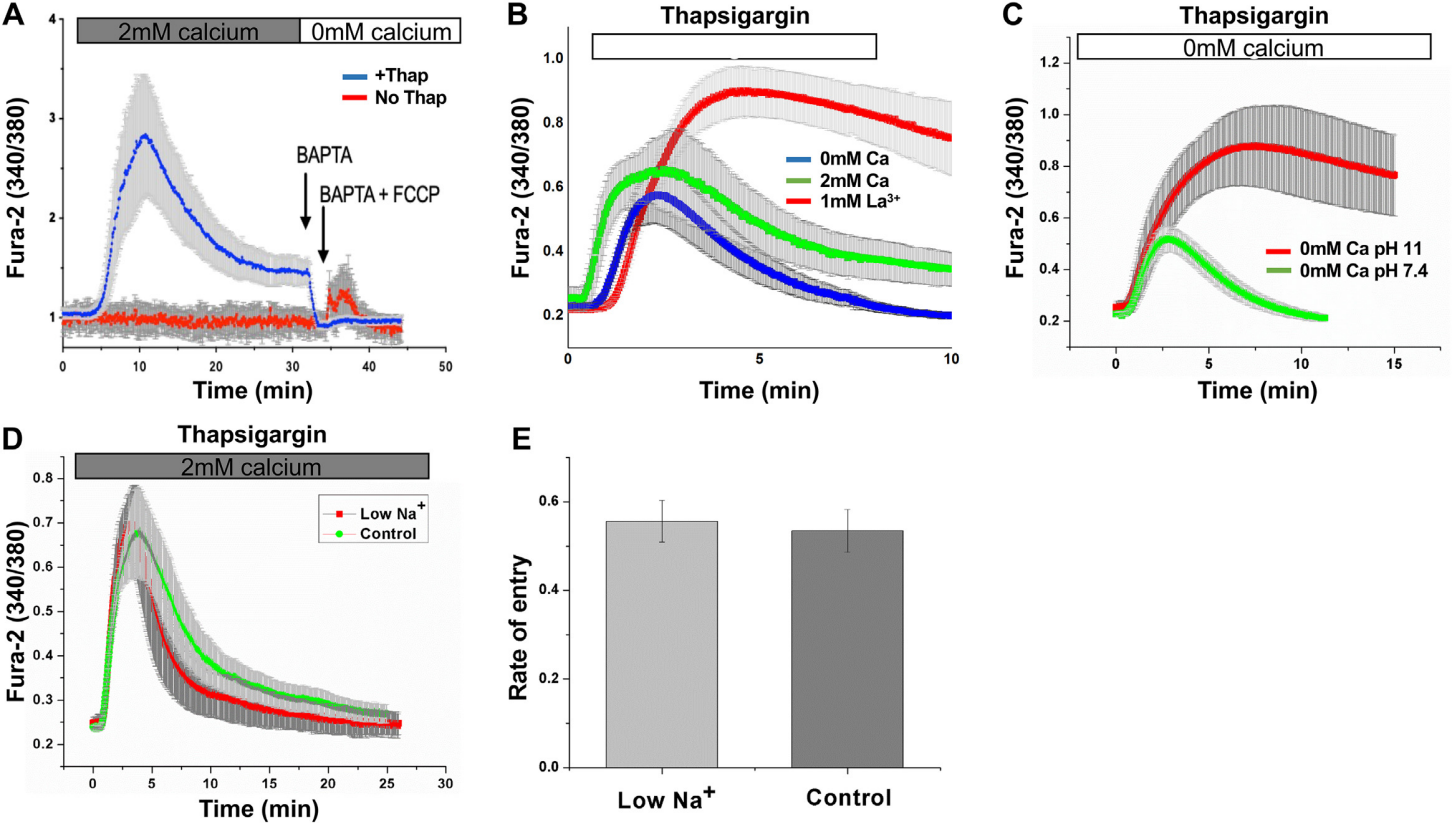
PMCA pump is the major Ca^2+^ clearance mechanism. (A) A small Ca^2+^ rise is evoked by FCCP both in control cells and after 15 min of stimulation with thapsigargin. (B) Block of the PMCA with external La^3+^ enhances the Ca^2+^ response to thapsigargin and delays its recovery. Data are mean of >20 cells for each condition. (C) The Ca^2+^ response is increased and sustained in alkaline pH, which inhibits the PMCA pump. Each trace is mean of >16 cells for each condition. (D) Removal of external Na^+^ has little impact on the recovery of cytosolic Ca^2+^ to thapsigargin. (E) Removal of external Na^+^ does not affect the half time for recovery of cytosolic Ca^2+^. *P* > 0.2 between the groups.

The inability of CRAC channels to raise bulk Ca^2+^ several minutes after their activation suggests the existence of a mechanism that effectively removes Ca^2+^ from the cytosol. Major Ca^2+^ clearance mechanisms in non-excitable cells include plasma membrane Ca^2+^ ATPase pumps and Na^+^–Ca^2+^ exchangers, SERCA pumps on the ER and mitochondrial Ca^2+^ uptake.^47^ A role for SERCA pumps can be excluded because we used thapsigargin to deplete stores. We assessed the contribution of mitochondria by comparing responses to the protonophore FCCP 15 min after stimulation with thapsigargin in the presence of external Ca^2+^, or after the same time but without thapsigargin treatment (control). No clear increase in cytosolic Ca^2+^ was seen when cells pretreated with thapsigargin were challenged with FCCP (Figure 6A). These data show that mitochondria had not accumulated cytosolic Ca^2+^ at this time, and therefore that another Ca^2+^ clearance mechanism was operational and which reduced the cytosolic Ca^2+^ rise evolved by CRAC channels. We focussed on plasma membrane Ca^2+^ transport.

The plasma membrane Ca^2+^ATPase pump is inhibited by milliMolar concentrations of La^3+^. Stimulation with thapsigargin in Ca^2+^-free solution supplemented with 1 m m La^3+^ led to a substantially larger and more prolonged cytosolic Ca^2+^ rise than was the case in either Ca^2+^-free or Ca^2+^-containing solution (Figure 6B). The larger increase in cytosolic Ca^2+^ following stimulation with thapsigargin in Ca^2+^-free solution in the presence of La^3+^ has been seen in other studies,^48,49^ and reflects the fact that the PMCA pump is the dominant Ca^2+^ removal mechanism under these conditions. Another property of the plasma membrane Ca^2+^ATPase pump is that transport activity is inhibited by alkaline pH because the transporter exchanges cytosolic Ca^2+^ for external H^+^.^50,51^ Stimulation with thapsigargin in Ca^2+^-free solution at pH 11 produced a much larger and more sustained Ca^2+^ response than at pH 7.4, consistent with a major role for the plasma membrane Ca^2+^ pump (Figure 6C). To address a role for Na^+^–Ca^2+^ exchange, we incubated cells in Na^+^-free external solution for 5 min prior to challenge with thapsigargin in Na^+^-free solution. The pattern of the Ca^2+^ signal was similar to that seen in Na^+^-containing solution (Figure 6D). Cytosolic Ca^2+^ declined slightly more quickly in Na^+^-free solution but no difference in the half times of decay of the Ca^2+^ signal was observed (Figure 6E). Collectively, these results identify a major role for plasma membrane Ca^2+^ ATPase pump in extruding Ca^2+^ that has entered through CRAC channels.

### Plasma Membrane Ca^2+^ ATPase Isoform 4b Accelerates Ca^2+^ Removal From the Cytosol

**Figure 7.**
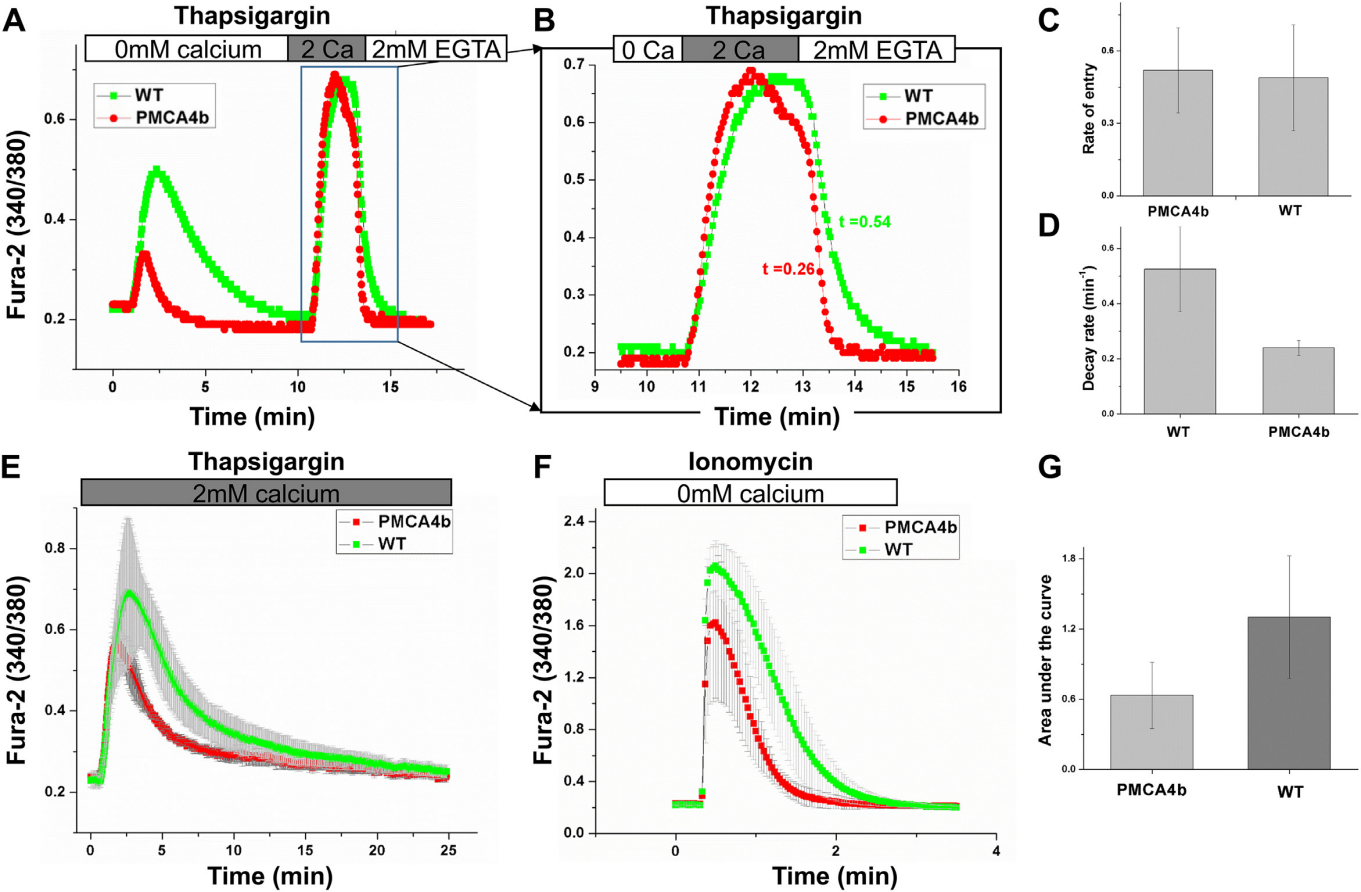
Overexpression of PMCA4b accelerates Ca^2+^ removal following store-operated Ca^2+^ entry. (A) The timecourse of cytosolic Ca^2+^ is compared for a control cell (mock transfected and labeled WT for wild type) and one expressing PMCA4b-cherry. (B) The box in panel A is shown on an expanded time scale. (C) The rate of Ca^2+^ entry from 22 cells is compared for the conditions shown. *P* > 0.3 between the two groups. (D) Aggregate data measuring the decay rate of the Ca^2+^ signal from 22 cells are compared for the conditions shown. Decay rate was measured as a linear fit to the initial steep decline of cytosolic Ca^2+^ when external Ca^2+^ was removed. *P* < 0.01 between the two groups. (E) Ca^2+^ signals are compared between wild type cells and cells expressing PMCA4b-cherry. Red trace is the mean of 22 cells and green trace of 26 cells. (F) Ca^2+^ signals to ionomycin challenge in Ca^2+^-free solution are compared for wild type cells and for cells expressing PMCA4b-cherry. Each trace is the mean of 16–21 cells. (G) Aggregate data measuring the area under the traces in panel E are compared. *P* < 0.05 between the two groups.

Mammalian plasma membrane Ca^2+^ATPase pumps are encoded by four separate genes.^23^ PMCA4b is a widely expressed member and is the major isoform found in HEK293 cells.^23^ If PMCA4b was responsible for removing Ca^2+^ entry through CRAC channels, a prediction would be that overexpression of the pump should lead to more rapid recovery of cytosolic Ca^2+^. Following expression of PMCA4b-cherry, we found that thapsigargin-evoked Ca^2+^ release was considerably smaller and more transient than in control cells (labeled WT; Figure 7A). Readmission of external Ca^2+^ led to a rapid rise in cytosolic Ca^2+^ due to store-operated Ca^2+^ entry (Figure 7A). The rate of Ca^2+^ entry was similar between cells expressing PMCA4b-cherry and mock-transfected control cells (Figure 7A; expanded in Figure 7B; aggregate data shown in Figure 7C). However, whereas control (WT) cells showed a transient plateau once cytosolic Ca^2+^ had peaked (Figure 7A, green trace), no plateau was evident in PMCA4b-expressing cells (Figure 7A, red trace). The subsequent decay rate of cytosolic Ca^2+^, measured in Ca^2+^-free solution, was considerably faster (∼2.5-fold) in the presence of recombinant PMCA4b (Figure 7D). Hence PMCA4b effectively removes Ca^2+^ that has entered the cytosol via CRAC channels. To examine whether increased expression of PMCA4b accelerated the decay of the cytosolic Ca^2+^ signal in the continuous presence of external Ca^2+^, we compared the pattern of the Ca^2+^ signal following thapsigargin stimulation between control cells and those expressing PMCA4b-cherry (Figure 7E). The cytosolic Ca^2+^ signal was smaller and returned to pre-stimulation levels ∼2-fold more quickly when the pump was overexpressed (Figure 7E).

To quantify the impact of PMCA4b overexpression on Ca^2+^ clearance, we stimulated cells with a high concentration of ionomycin (2 μm) in the absence of external Ca^2+^ and compared the duration of the cytosolic Ca^2+^ rise (measured as the area under the curve) between control cells and those overexpressing PMCA4b. The Ca^2+^ signal was smaller and more transient when PMCA4b was overexpressed (Figure 7F and G).

### Relative Distribution of Orai1 and PMCA4b

**Figure 8.**
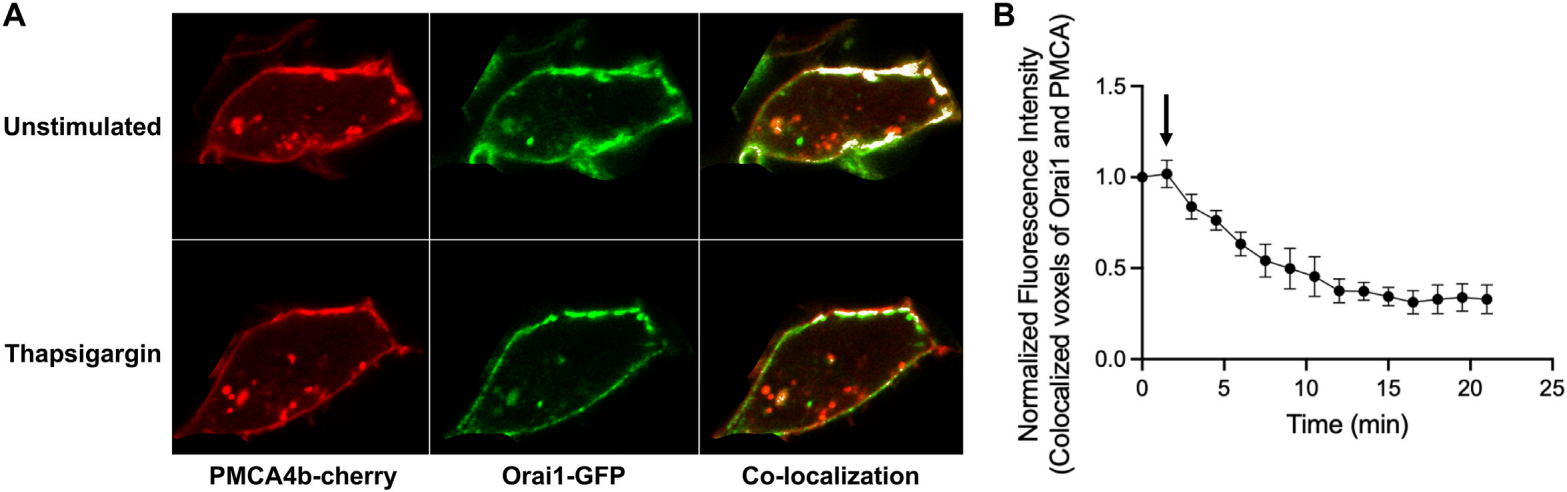
Airy scan confocal microscopy shows reduced co-localization of PMCA4b and Orai1 after stimulation. (A) Images compare distribution of Orai1-GFP and PMCA4b-cherry under resting conditions (upper) and after thapsigargin treatment for 20 min (lower). Computed co-localization is indicated in white. (B) Graph shows the change in colocalized voxel of Orai1 and PMCA4b after exposure to thapsigargin. Each point after 2 min (arrow) was significantly different from the value at 2 min. *P* < 0.05 for the first two points and then *P* < 0.01 for the rest. Data are presented as mean ± SEM.

We used Airyscan confocal microscopy to assess the relative spatial distribution of PMCA4B-cherry and Orai1-GFP. In unstimulated cells, both proteins were distributed throughout the cell periphery and this led to significant overlap in their distribution (Figure 8A; upper panel; co-localization is shown in white in the right hand image). Interestingly, PMCA-cherry was also contained in numerous relatively small cytosolic vesicles, and to a much greater extent that Orai1-GFP. Many vesicles seemed to contain only one type of tagged protein. After stimulation with thapsigargin in the presence of external Ca^2+^, PMCA distribution changed little if at all, whereas Orai1 formed clusters (Figure 8A, lower set of images). The extent of overlap was now considerably less (aggregate data are summarized in Figure 8B). These results suggest that Orai1 does not interact physically with PMCA4b. The overlap in distribution in resting cells reflects the homogenous distribution of both proteins in the plasma membrane, with some overlap by chance. However, after store depletion, Orai1 clusters at endoplasmic reticulum–plasma membrane junctions and, in the absence of a direct interaction between Orai1 and the PMCA4b, the extent of spatial overlap decreases as the pump does not accumulate at these sites.

## Discussion

The widely expressed PMCA4b isoform is considered a house keeping PMCA, maintaining low cytosolic Ca^2+^ in resting cells and thereby preserving a concentration gradient for Ca^2+^ entry. Our new results demonstrate the versatility of PMCA4b in shaping Ca^2+^ signals evoked by store-operated Ca^2+^ entry. Remarkably, we find the activity of PMCA4b adjusts over time to overcome continuous store-operated Ca^2+^ entry and return bulk cytosolic Ca^2+^ to resting levels despite a prolonged increase in membrane permeability to Ca^2+^. Because store-operated Ca^2+^ entry takes several seconds to initiate and then develops over tens of seconds when stores are depleted passively with thapsigargin, the initial large increase in cytosolic Ca^2+^ followed by its recovery is consistent with the finding that modulation of PMCA is acquired slowly,^25^ taking several tens of seconds to adapt to a new level of activity.

It is noteworthy that cytosolic Ca^2+^ decayed back to resting levels in native HEK293 cells despite continued Ca^2+^ entry through CRAC channels. In Jurkat T cells, the Ca^2+^ signal stabilized at an elevated plateau^25^ and a similar profile was seen in RBL cells (Supplementary Figure 2 in^52^). In all three cell types, PMCA4b is expressed. Why therefore is the Ca^2+^ signal relatively transient in HEK cells? Two factors need to be considered: First, the number of functional CRAC channels and second, the number of active PMCA pumps along with the rate and extent of their modulation. The relative levels of expression of the pump in the different cell types are unknown. However, CRAC channel density is markedly different. Jurkat T lymphocytes and RBL cells exhibit large native CRAC currents, of ∼−15 and −40 pA at −80 mV (both equating to ∼−3 pA/pF). By contrast, the native CRAC current in HEK cell is generally too small to be measured accurately. Since the level of detection of a whole cell current in a cell the size of a HEK cell is ∼−1.5 pA, we assume the endogenous HEK cell macroscopic CRAC current is ≤1.5 pA. Taking a typical non-excitable cell buffering capacity of ∼100 and ignoring Ca^2+^-dependent inactivation, which would be modest over the physiological range, CRAC current would raise cytosolic Ca^2+^ in a HEK cell at a rate of ∼50 n m/s. By measuring the initial fast phase of decay of cytosolic Ca^2+^ on switching to Ca^2+^-free solution after Ca^2+^ entry through CRAC channels for 4 min, we estimate Ca^2+^ clearance by PMCA in HEK293 cells to be ∼25 n m/s, only 2-fold less than that reported in Jurkat T cells.^25^ Since PMCA modulation develops over minutes, pump activity will develop sufficiently to match the rise in cytosolic Ca^2+^ achieved by CRAC channel opening in HEK cells. Therefore, the main reason why cytosolic Ca^2+^ declines fully in HEK293 cells and only partially in Jurkat T lymphocytes most likely reflects the much larger number of functional CRAC channels in the latter cell type. Although our estimate of the rate of rise of cytosolic Ca^2+^ due to CRAC channel activity is not derived from empirical measurements but is based on calculations that rely on several assumptions such as the Ca^2+^ buffering capacity of the cytosol, the cytosolic volume and a reasonable estimate of the Ca^2+^ current, it is not dissimilar from the rate of Ca^2+^ removal by PMCA, suggesting the processes can be matched.

Our experiments in HEK293 cells are in good agreement with studies in Jurkat T cells that show PMCA4b activity remains high during store-operated Ca^2+^ entry.^25,53^ By contrast, STIM1 has been reported to inhibit PMCA4b in Jurkat T cells, dramatically slowing down Ca^2+^ clearance.^26^ This effect was attributed to the N-terminus of STIM1 because a construct truncated immediately after the proline-rich region, STIM1Δ597, no longer pulled down with PMCA in co-immunoprecipitation experiments and did not slow Ca^2+^ removal. The effect of STIM1 on PMCA has been been reported to depend on the presence of the protein POST. Overexpression of POST in Jurkat T cells prevented STIM1 from inhibiting PMCA4 and so Ca^2+^ entry through CRAC channels was cleared rapidly from the cytosol. Knockdown of endogenous POST slowed Ca^2+^ clearance.^27^ These findings conflict with an earlier study by Clapham and colleagues who showed, also in native Jurkat T cells, that siRNA-dependent knockdown of POST increased Ca^2+^ clearance, leading to the conclusion that POST served as an inhibitor of PMCA.^54^ These authors also found that POST was critical for STIM1 to bind to and inhibit PMCA.^54^ Further work is needed to reconcile these contradictory reports on whether and how STIM1 and POST affect PMCA activity in Jurkat T cells.

We took advantage of the genetically encoded GECO fluorescent Ca^2+^-sensitive protein to measure Ca^2+^ levels adjacent to CRAC channels and in the bulk cytosol. Whereas bulk Ca^2+^ returns to pre-stimulation levels within a few minutes of sustained Ca^2+^ entry through CRAC channels, Orai1-GECO continued to report elevated Ca^2+^ near the channels for several minutes. Similar results were obtained when we used TIRF microscopy to measure local Ca^2+^ near CRAC channels. These findings confirm that CRAC channels remain active despite bulk Ca^2+^ returning to resting levels and highlight the presence of stable, standing Ca^2+^ gradients between CRAC channels and bulk cytosol. Using a lipid-modified Fura 2 analogue that measures Ca^2+^ near membranes, Go et al. reported that sub-membranous Ca^2+^ could be reduced by overexpression of PMCA4b whereas bulk Ca^2+^ increased under the same conditions in jurkat T cells.^27^ Our direct measurements of Ca^2+^ adjacent to CRAC channels confirm that local and bulk Ca^2+^ signals are markedly different; local Ca^2+^ near the channels remains elevated despite bulk Ca^2+^ returning to pre-stimulation levels.

To develop an appropriate immune response, T lymphocytes require prolonged Ca^2+^ entry through CRAC channels as well as the formation of an immunological synapse with the antigen-presenting cell. STIM and Orai1 are recruited to the synapse, resulting in long-lasting local Ca^2+^ influx.^55^ Quintana et al. have demonstrated that mitochondria also accumulate at the immunological synapse and efficiently buffer Ca^2+^ entry through CRAC channels, outcompeting PMCA4b for Ca^2+^ removal.^53^ Mitochondria then release Ca^2+^ deeper into the cytosol through their Na^+^–Ca^2+^ exchanger, resulting in an elevated bulk cytosolic Ca^2+^. In HeLa cells, where mitochondria do not actively accumulate near CRAC channels, stimulation with thapsigargin evoked high local Ca^2+^ but a small bulk Ca^2+^ rise.^53^ PMCA pump activity in HeLa cells therefore matches Ca^2+^ entry through CRAC channels to ensure low global Ca^2+^, as we describe in HEK293 cells.

PMCA pumps relocate to the immunological synapse following T cell receptor stimulation. Detailed modelling studies have shown that the redistribution of PMCA pumps to the synapse results in lower local Ca^2+^ but a larger sustained global rise.^56,57^ A more homogeneous distribution of the pump, as occurs with thapsigargin stimulation, leads to a considerably lower global Ca^2+^ rise but a larger local Ca^2+^ signal below the plasma membrane. These conclusions are in excellent agreement with our experimental findings. Our Airy scan confocal microscopy data confirm an earlier study by Quintana et al., who also showed that PMCA4b and Orai1 did not colocalize in T cells either before or after stimulation with thapsigargin.^53^ As predicted by Hoth and colleagues, the relatively homogeneous distribution of PMCA4b pumps compared with Orai1 channels led to a high and sustained local Ca^2+^ but a much smaller and transient global Ca^2+^ signal.

Due to the lack of co-localization between PMCA4b pumps and CRAC channels, PMCA4b is unlikely to impact on the size or lateral extent of the Ca^2+^ nanodomain near each hexameric CRAC channel, but instead will reduce spillover or smearing of the Ca^2+^ signal at the edges of ER–PM junctions, thereby preventing local Ca^2+^ entry from developing into a more global Ca^2+^ rise. The more homogeneous dispersal of the pump will also ensure Ca^2+^ is removed throughout the cytosol, thus helping maintain a low global rise. Because a bulk rise in cytosolic Ca^2+^ triggers Ca^2+^-dependent slow inactivation of CRAC channels, the lowering of cytosolic Ca^2+^ by the PMCA will reduce inhibition of CRAC channels and thus, sustain Ca^2+^ entry. Furthermore, by sharpening the local Ca^2+^ signal, PMCA4b ensures the specificity of the local Ca^2+^ signal is maintained by restricting activation of signalling pathways to within the realm of the Orai1 Ca^2+^ nanodomain.

## Data Availability

The data underlying this article will be shared on reasonable request to the corresponding author.
